# Fabricating Pyramidal Lattice Structures of 304 L Stainless Steel by Wire Arc Additive Manufacturing

**DOI:** 10.3390/ma13163482

**Published:** 2020-08-07

**Authors:** Haorui Zhang, Junjin Huang, Changmeng Liu, Yongsheng Ma, Yafeng Han, Tianqiu Xu, Jiping Lu, Hongli Fang

**Affiliations:** 1 School of Mechanical Engineering, Beijing Institute of Technology, Beijing 100081, China; 3120180422@bit.edu.cn (H.Z.); 15600920528@163.com (J.H.); liuchangmeng@bit.edu.cn (C.L.); Xutianqiu@hotmail.com (T.X.); jipinglu@bit.edu.cn (J.L.); fanhongli2000@sina.com (H.F.); 2 Department of Mechanical Engineering, University of Alberta, Edmonton, AB T6G2H5, Canada; yongsheng.ma@ualberta.ca

**Keywords:** wire arc additive, manufacturing, lattice structures, 304 L stainless steel, microstructure

## Abstract

Lattice structures have drawn considerable attention due to their superior mechanical properties. However, the existing fabrication methods for lattice structures require complex procedures, as they have low material utilization and lead to unreliable node connections, which greatly restricts their application. In this work, wire arc additive manufacturing is used to fabricate large-scale lattice structures efficiently, without any air holes between rods and panels. The principle and the process of fabricating the rods were analyzed systematically. The influence of the two most important parameters, including heat input and preset layer height, is disclosed. Through optical microscopy, the microstructure of the fabricated steel rods is found to consist of dendritic austenite and skeletal ferrite. The tensile strength of the rods can reach 603 MPa, and their elongation reaches 77%. These experimental results demonstrated the feasibility of fabricating lattice structures using wire arc additive manufacturing.

## 1. Introduction

With the rapid development of material processing and machining technologies, large-scale lattice structures had drawn considerable attention in many applications wherein mechanical performance and weight-saving are essential, such as the aerospace, military and construction industries [1,2,3,4,5]. According to different requirements, the mechanical and functional properties of lattice structures can be designed through their relative density, core morphology, materials and inclusions [6].

However, till now, large-scale lattice structures have not been widely used. The main reason is that there is no efficient processing method for complex surfaces. The traditional fabrication methods of large-scale structures mainly include investment casting, slotted interlocking and punched hole mesh drawing [7]. The investment casting process involves the high cost of manufacturing moulds, and is prone to defects. Moreover, due to the limitation of metal fluidity, the investment casting method struggles to manufacture low-density lattice structures with complex structures [8]. Slotted interlocking is characterized by complex processes, long lead time and high manufacturing costs, which limits the development of its practical applications [9,10]. The punched hole mesh drawing method has the problems of a low utilization ratio of raw material, a complex working procedure and the limitations of material selection [11,12]. What is more, all these methods also need to connect the lattice core and face sheets. The research of Ming et al. [13] shows that the fracturing of the nodes directly leads to the failure of the lattice sandwich panel. At present, the connection between metal lattice cores and face sheets is mainly through welding [14], but the nodes undergo stress concentration and residual stress, which makes them prone to fracture [15]. As has been noted, due to the various deficiencies of the traditional methods, we urgently need to develop a much leaner and much more efficient method for fabricating large-scale lattice structures and promoting their practical application.

Additive manufacturing (AM) is a novel fabrication technology, based on Computer Aided Design model data, which builds near-net-shape parts layer by layer [15,16]. Unlike traditional subtractive manufacturing, where the material is removed through cutting, boring, drilling and grinding [17,18], AM is a “bottom-up” manufacturing method based on the principle of the gradual accumulation of materials, which means that it has unprecedented flexibility of design, can achieve a high utilization of raw material, and is much more efficient. Selective laser melting (SLM) is the most-used AM process, and builds parts through melting metal powders selectively with computer-aided design (CAD) data [19]. However, due to its low deposition rate and high cost, this method is only suitable for small-sized structures [20].

On the other hand, wire arc additive manufacturing (WAAM) is a variant of AM that is classified in the category of direct energy deposition, according to ASTM F2792-12a [21]. WAAM uses an electric arc as the heat source, a metal wire as the feedstock material and a computerized numerical control (CNC) system to form the desired geometry. Compared with SLM, WAAM has a much higher deposition rate and lower costs [22]. Therefore, WAAM is quite suitable for the large scale components of complex geometry. However, the volume of the molten pool in arc additive manufacturing is large, and the existence of disturbance factors, such as cold raw materials and arc force during the forming process, makes the pool an unstable system. These reasons make it difficult for WAAM to produce complex structures, and related research is rare. Only recently, Abe and Sasahara [23] used WAAM based on melt inert-gas (MIG) welding to build lattice structures, and investigated the influences of some process parameters. Different from their work, our lab developed the tungsten inert gas (TIG) WAAM, which has a more stable pulse [24,25].

In this paper, we further study the TIG WAAM and use it to successfully fabricate large-scale pyramidal lattice structures made of 304 L stainless steel. The technical processes of fabricating rods and their connections to the panel are studied. The principle and process were analyzed systematically, and the influences of important process parameters were investigated meticulously. The preparation, morphology, microstructure and mechanical properties of the fabricated parts were derived as well.

## 2. Manufacturing Methods 

### 2.1. Experimental Equipment and Material

**Figure 1:**
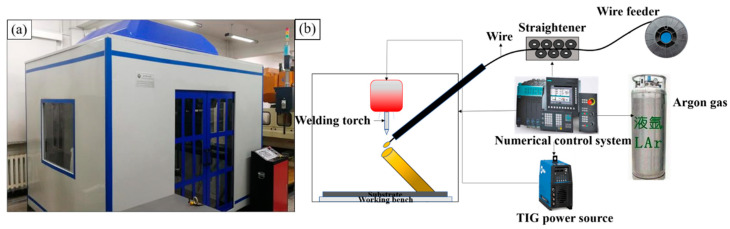
(**a**) Overview of wire arc additive manufacturing equipment; (**b**) schematic diagram of wire arc additive manufacturing process.

The manufacturing of specimens was carried out using the large-scale (WAAM) instrument self-developed by the Beijing Institute of Technology, the schematic and actual equipment of which are shown in Figure 1. The experiment setup mainly consists of a three-axis CNC machine tool, gas tungsten arc welding (GTAW) equipment (Dynasty 350; Miller, Appleton, WI, USA), a computer, a wire feeder system (WEILD, Guangzhou, China), a three-dimensional workbench and working chamber. The CNC controls the position and travel speed of the nozzle with a pre-written G code. The wire feeding system includes a wire feeder that controls the wire feeding speed and a wire straightener that reduces the curvature of the wire. The wire used for the study is 304 L stainless steel of 1.2 mm in diameter, which is a kind of austenitic stainless steel, and Guang et al. reported that it performs well in high temperatures with no heat treatment hardening phenomenon [26]. The chemical composition of the wire is listed in Table 1. The rolled 304 L stainless steel plate, with a size of 150 mm × 150 mm × 5 mm (width × length × height), was used as the substrate.

### 2.2. Manufacturing Principle

**Figure 2:**
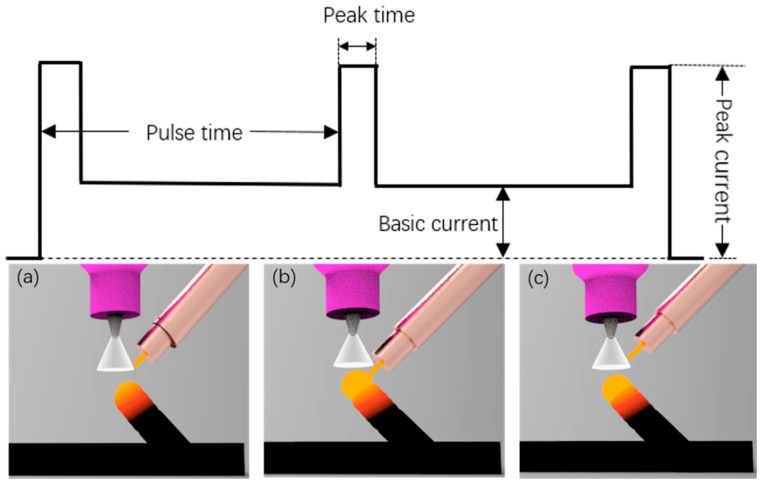
Deposition process of wire arc additive manufacturing of (**a**,**c**) the droplet shapes in the base current period, and (**b**) droplets transfer during peak current period.

Compared with existing AM methods, WAAM is an arc welding process based on tungsten inert gas arc welding (TIG). The arc formed between a pointed tungsten electrode and a substrate in an inert atmosphere of argon is used as the heat source. The arc current is pulsating in a direct current, and its waveform is shown in Figure 2. As shown in Figure 2b, the wire is melted and forms a liquid weld metal drop adhering to the wire in the base current period. While in the peak current period, the molten pool is transferred from the welding wire to the layer under the influence of droplet impact, arc power and gravity. Then the molten pool solidifies quickly within the base current time of the next pulse cycle, and the next wire is melted in this period, thus repeating the cycle. Through repeated spot-welding processes, rods can be formed.

### 2.3. Manufacturing Process

Firstly, a steel wire grinding wheel machine is used to polish the substrate fixed on the working bench with some countertop clamps. Then, we start the arc to wet the substrate. The preheating parameters are listed in Table 2. In fact, the welding torch is in place and the arc heats the substrate for three cycles without feeding the wire. After the preheating, the wire is fed at a constant speed and melted in the base current period with burning back, then a droplet will be formed at the end of the wire and is transformed to the substrate with the arc blow force and feeding momentum in the peak current period. Then the welding torch fixed on the guide rail beam of the computer numerical controlled gantries moves a certain distance along the set path. At the same time, the molten pool solidifies quickly in the base current time of the next pulse cycle and the new coming wire is melted, repeating the cycle. All of this forms a cycle of “Feed–Melt–Transfer–Move”, and the droplets solidify layer by layer and form continuously in a fixed direction with the movement of the welding torch. 

**Figure 3:**
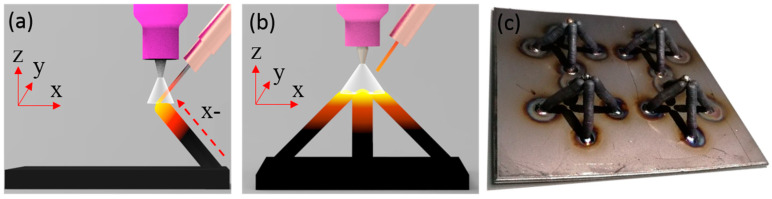
(**a**) The schematic diagram of manufacturing process of x−; (**b**) the schematic diagram of one cell; (**c**) the sample manufactured by WAAM.

**Figure 4:**
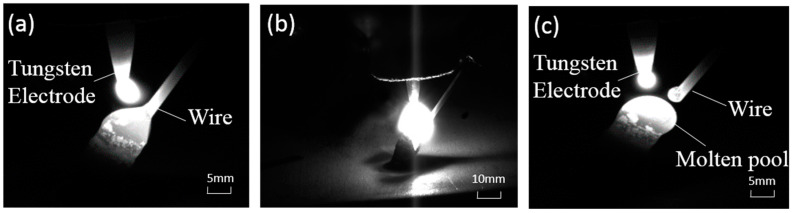
Actual image of layer accumulation. (**a**) wire feeding; (**b**) peak time; (**c**) completing the transition.

After completing the manufacturing of a single rod, we need to move the welding gun and complete the manufacturing of the remaining three rods in the same way, as shown in Figure 3a,b. In this process, we need to determine the manufacturing sequence of the inclined rods according to whether there is any interference in space. Because the wire feed tube is on the right side of the welding gun, the printing sequence can be as follows: X−, Y−, X+, and Y+. Figure 3c shows the sample manufactured by this process. The three images shown in Figure 4 were captured by a CCD camera (avA1900-50gm, Basler, Ahrensburg, Germany), which show the deposition process visually.

### 2.4. Experiment Detail

In this study, the key was to investigate the influence of process parameters, and find the relationship between molding defects and process parameters, exploring the optimum combination of parameters. The parameters we can adjust are peak current Ip [A], base-to-peak current ratio α [%], peak time ratio β [%], frequency f [HZ] and layer height Hs [mm]. The heat input Q can be calculated using the following equation [27]:

(1) $$Q={\eta I}_{\mathrm{av}}\frac{U}{T_{m}}$$

(2) $$I_{\mathrm{av}}=\frac{I_{p}T_{p}+I_{b}T_{b}}{T_{p}+T_{b}}$$

where U is the average voltage of the TIG welding, I_av_ is the average current, T_m_ is the torch moving speed, I_p_ is the peak current, I_b_ is the base current, T_p_ is the peak time, T_b_ is the base current duration and η is the arc conversion efficiency (assumed to be 0.7). Additionally, during the whole process, we use argon gas with a purity of 99.99% as the shielding gas, and the gas flow rate was 20 L/min. On the basis of a large amount of experimental experience in the early stage, we generally set the distance between the tungsten electrode and the substrate to 8 mm, and the distance between the tungsten electrode and the feed wire to 6 mm, which can ensure stable arc starting and arc impact.

With a lot of tests for building inclined rods, we found that the preset layer height and heat input are two of the most important process parameters. On the basis of the preliminary basic research, four sets of experiments were set up. The preset layer height was changed to observe the appearance of rods; the specific parameters are listed in Table 3. The preset layer height is actually the Z direction displacement. Then, four groups of tests with different heat input, Q, were conducted to explore the influence of the heat input. The heat input Q was changed by varying Ip and β. In all the experiments, the frequency f was always 0.5 HZ, while the torch moving speed Tm was 10 mm/s. The specific parameters are shown in Table 4. In order to eliminate the influence of the layer height, the layer height here, including the first five layers Ho and the rest Hr, was determined by observing the actual molten pool height using CCD, to ensure that the molten pool did not squeeze the previous drop of molten pool, and that the next drop of molten pool can also smoothly transition to the previous drop of molten pool so as to complete the overlap. The dimensions of the inclined rods’ diameters were measured by vernier caliper, with an accuracy of 0.02 mm. The actual layer height was calculated by dividing the total height by the actual number of the depositing layers. The measurement values were averaged.

**Figure 5:**
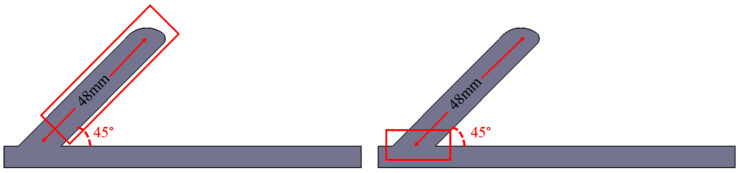
Diagram of metallographic samples’ positions.

In order to analyze the structure of the rods and the connection quality between the rods and the face sheets, the microstructure characteristics were observed by optical microscopy (Leica DM4000M; Leica, Solms, Germany). The metallographic samples were taken according to the position shown in Figure 5. The metallographic specimen was treated via standard mechanical polishing, and etched with a solution of 10 mL HNO_3_ (nitric acid), 30 mL HCL (hydrochloric acid) and 20 mL glycerol for about 40 s. After that the microstructure was characterized with optical microscopy (Leica DM4000M; Leica, Solms, Germany).

**Figure 6:**
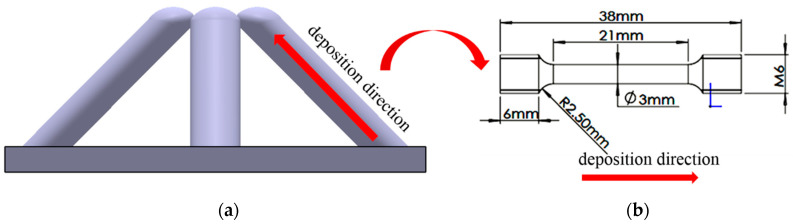
(**a**) Schematic illustration of the way to take the metallographic specimen; (**b**) size of the tensile test sample.

In order to understand the mechanical properties of the rods made by WAAM, a room temperature uniaxial tensile test was conducted. Three inclined rods fabricated by the optimum parameters that are shown in Table 3 were made to the size shown in Figure 6b. The angles of the inclined rods are all 45° and the lengths are 48 mm, as shown in Figure 5. These experiments were carried out on an Instron 5966 electronic universal material testing machine. Here, the axial direction of the specimens was parallel to the deposition direction, and the columnar specimens were processed into the tensile specimen with Φ 3 mm, as shown in Figure 6. Room-temperature tensile properties were tested according to the test standard of ISO 6892-1:2009 [28]. In order to ensure the accuracy of the measured data, we took three diagonal bars for the tensile test. The fracture surface was analyzed by the BCPS4800 scanning electron microscope (SEM; BCPS, Baltimore, ML, USA).

## 3. Results and Discussion

### 3.1. Influence of Heat Input and Preset Layer Height on Rod’s Appearance

**Figure 7:**
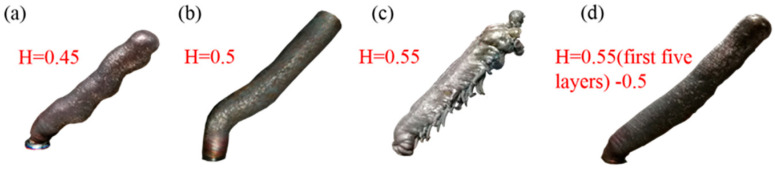
Four typical rods with different preset layer height; (**a**) the typical sample morphology with 0.45 mm preset layer height; (**b**) the typical sample morphology with 0.5 mm preset layer height; (**c**) the typical sample morphology with 0.55 mm preset layer height; (**d**) the typical sample morphology with 0.55 mm preset layer height in the first five layers, which then becomes 0.5 mm.

**Figure 8:**
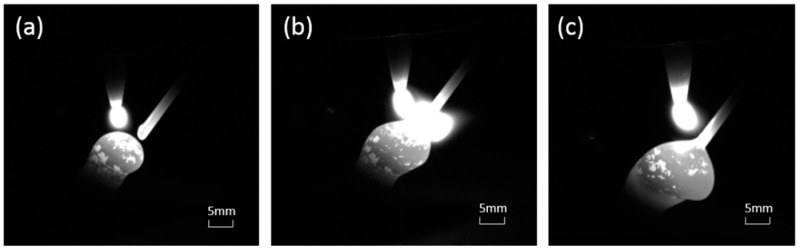
CCD images of the manufacturing process with a too-low layer height: (**a**) wire feeding; (**b**) peak time; (**c**) the droplet is squeezed and bent.

**Figure 9:**
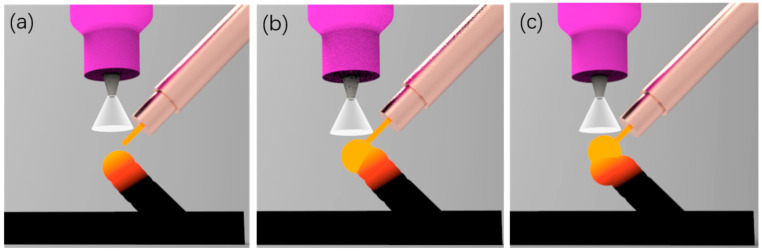
Schematic of manufacturing process with too-low layer height. (**a**) wire feeding; (**b**) peak time; (**c**) the droplet is squeezed and bent.

Figure 7 is the rods built with the same experimental parameters, except for preset layer height that is listed in Table 2. As can be seen from Figure 7a, when the preset layer height is 0.45 mm, the rod is twisted. Unlike the distortion caused by excessive heat input, we can clearly see that the distortion of the rod in Figure 7a is more like a periodic bending. Through observation, we determined that this is because the actual layer height of the molten pool is higher than the preset layer height. Figure 8 shows the CCD images of this process. The new molten pool does not have enough space, so it is squeezed and then bent. After bending, the new coming molten pool has enough space to grow normally, but the next drop does not have enough space, and then bending occurs again. Repeatedly, a twisted rod appears, as shown in Figure 7a. Figure 9 is the schematic diagram of this process. As shown in Figure 7b, when the layer height is 0.5 mm, the overall forming quality of the inclined rod is good, however the part close to the substrate does not grow in the set 45° direction, but close to the right angle. Through analysis, we think this is due to the cooling effect of the substrate, according to Young’s equation:

(3) $${\cos\theta \gamma }_{l-g}=\gamma_{s-g}-\gamma_{l-s}$$

**Figure 10:**
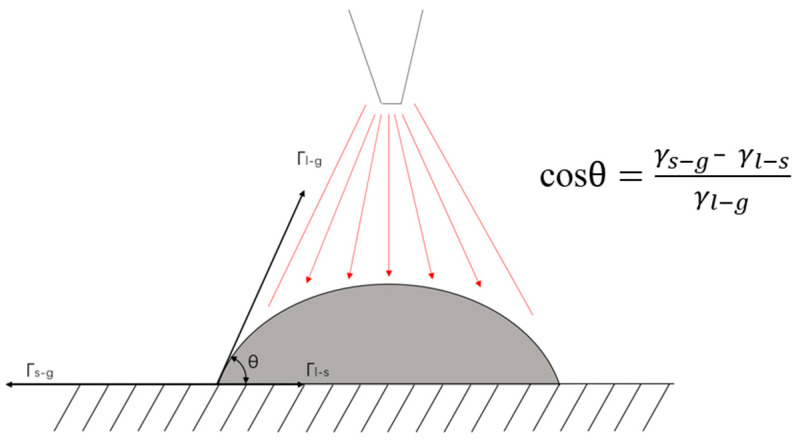
Schematic diagram of the relationship between the spread of the molten pool and the contact angle.

**Figure 11:**
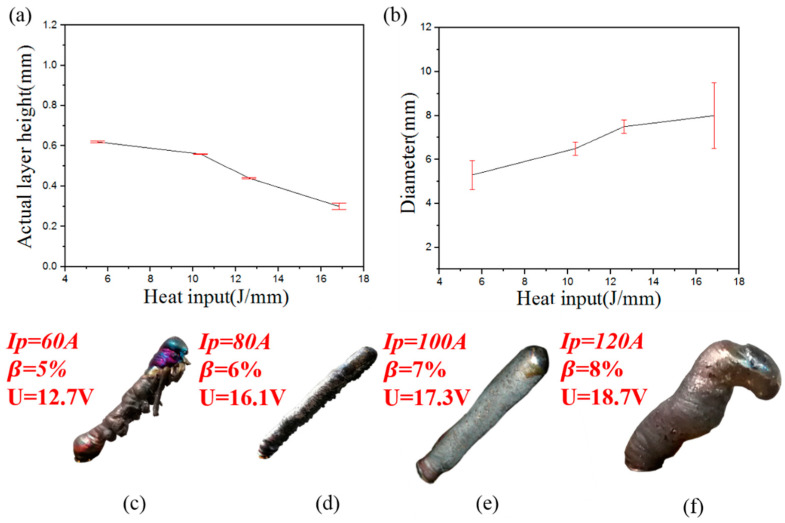
(**a**) Relationship between heat input and actual layer size; (**b**) Relationship between heat input and diameter; (**c**) Typical sample morphology in group I; (**d**) Typical sample morphology in group II; (**e**) Typical sample morphology in group III; (**f**) Typical sample morphology in group IV.

**Figure 12:**
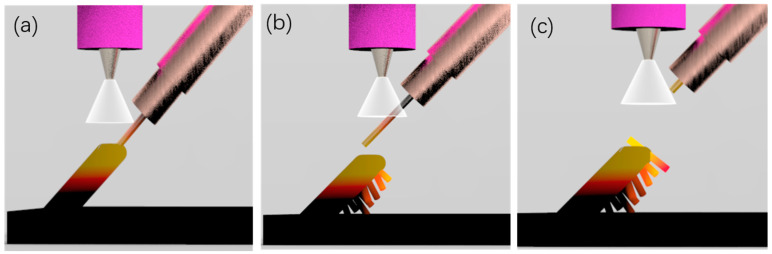
Schematic of manufacturing process with too-high layer height. (**a**) wire feeding; (**b**) Lap failure; (**c**) Peak current knocks down the wire.

Due to the heat dissipation effect of the substrate, the temperature of the droplets closer to the substrate is lower, and the surface tension γ_l−g_ of the molten pool is larger, resulting in the increase of the contact angle θ, the smaller diameter and the higher actual layer height. Figure 10 is the schematic diagram of the relationship between the spread of the molten pool and the contact angle. The movement of the welding torch is controlled by a pre-written G code. In the X+ direction, the movement code is X+ 0.5, Z+ 0.5. Therefore, the next drop of the molten pool will be inserted into the previous drop, and under the action of large surface tension, it will shrink to the previous molten pool. After a few layers, the heat dissipation effect of the substrate on the molten pool is gradually weakened. Then the rod can be built at the set 45°. As Figure 7c shows, when the preset layer height is 0.55 mm, there will be a lot of unmelted wire on the back of the rod, but no unmelted metal wires on the side of the rod near the arc. This is different from the unmelted wires caused by low heat input, that were shown in Figure 11c. As shown in schematic Figure 12, when the layer height is too high, the wire cannot stick in the molten pool and complete the lap. Then the wire easily penetrates through the welding arc column area, and is burned in sections, which also interferes with the arc voltage, causing the arc length to be disordered and affecting the continuous forming of the rod. The wire near the side of the arc will melt under the action of the arc. The last group was to set the height of the first five layers: to 0.55 mm, and then to 0.5 mm. As is shown in Figure 7d, the part of the rod closest to the substrate did not show the phenomenon shown in Figure 7b, but grew in the set 45° direction According to the above-mentioned analysis, we know that the preset layer height should be strictly controlled to match the actual layer height. Only when the displacement of the welding gun in the Z direction per unit of time is equal to the height of the molten pool can the deposition be carried out stably, and we can get a good formed rod.

**Figure 13:**
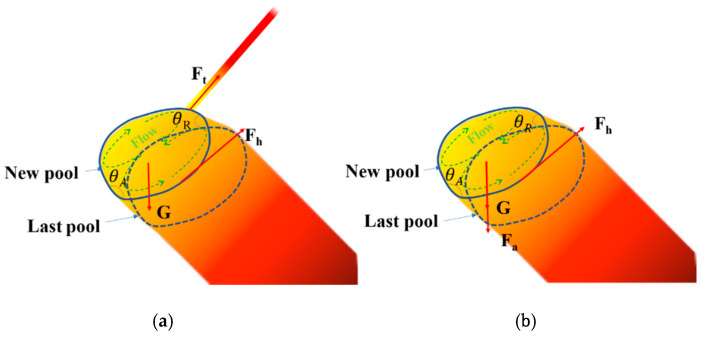
(**a**) Diagram of force analysis during wire feeding; (**b**) Diagram of force analysis during molten pool transition.

As can be seen from Figure 11, the fabrication of stable rod shapes depends on the heat input Q. The actual layer height decreases with the pace of increased heat input, while the diameter increases with the increase in heat input. Four typical types of inclined rods appeared under different heat conditions in Figure 11. As is shown in Figure 11c, when the heat input is low, that is, Q < 5.53 J/mm, the wire material cannot melt completely, and adhesion is caused. The diameter is about 5.24 mm, which is related to the stress condition of molten pool. As is showed in Figure 13, the force of the molten pool can be divided into dispersion force and coupling force. The main dispersion force is the welding arc force Fa and gravity of the molten pool liquid metal G [29], while the main coupling force is the surface tension Ft. Different from the contact angle model in Figure 10, θ_A_ is greater than θ_R_. The difference between θ_A_ and θ_R_ is called contact angle hysteresis, and the hysteresis force Fh is produced due to the existence of contact angle hysteresis. The hysteresis force is equal to Ftω × (cosθ_R_ − cosθ_A_) [29]. Here, ω is the width of the droplet. The arc force Fa is mainly related to the current, voltage and pulse frequency. When the heat input is low, which refers to the peak current and peak time, the Fa is too small to transfer the droplet from the metal wire, resulting in the adhesion and small diameter. As the heat input increases, the arc force Fa becomes larger. In the same volume of molten pool, the molten pool is more impacted and the diameter will be greater, which is consistent with the experimental results in Figure 11. When the heat input Q > 16.83 J/mm, the molten pool cannot yet solidify before the new molten pool arrives, and it will collapse, for the gravity G exceeds the hysteresis Fh.

### 3.2. Microstructure Characterization

A complex thermal cycle occurs in the WAAM process and the thermal cycling process if each of the cladding layers is not the same. Karlsson et al. showed that the main solidification mode of 304 L Austenitic Stainless Steel in the molten pool is FA mode, that is to say, the primary phase is δ-Fe at the beginning of solidification, and then the solid-state transformation occurs after the reaction of peritectic and eutectic; δ becomes γ, and transforms into austenite [30]. In the following figures, black is the ferrite phase and orange is the austenite phase.

#### 3.2.1. Microstructure of Rod

**Figure 14:**
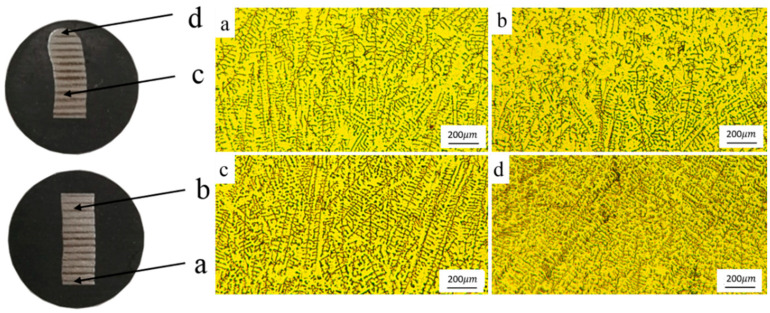
Longitudinal section of the inclined rods and microstructure of different positions of inclined rods.

Figure 14 shows the longitudinal section of the inclined rods and the microstructure. From the top of the rod to the substrate, four regions were selected for observation of their characteristics. We can clearly see the layer bands showing that the inclined rod is formed by the layer solidification of droplets. Zone a is a transitional area, and we can see from Figure 14a that there is a lot of austenite and a little ferrite in this area. Because this area is close to the substrate and the substrate has strong heat dissipation, the solidification speed of the droplets and the cooling rate of the solid phase transformation are very fast. When the ferrite is transformed into austenite, the compositions of Cr, Ni and other alloys at the ferrite–austenite grain boundary cannot fully diffuse, and the composition segregation is serious, resulting in the supercooling of the composition and less ferrite transformation. Zone b is the fusion zone between the coating layers, in which the microstructure is shown as a columnar austenite crystal with residual linear ferrite at its grain boundary. During the solidification of the liquid metal in the cladding layer, because the heat dissipation is the fastest in the direction perpendicular to the fusion line, the temperature gradient is larger and the undercooling is larger. Columnar ferrite grows along the direction perpendicular to the fusion line, and the columnar ferrite changes into columnar austenite and residual linear ferrite with the decrease in temperature. As the distance from the fusion line increases, the supercooling decreases and the component supercooling increases. The ferrite dendrites grow in the liquid–solid phase transition, and the residual dendrites are obtained in the solid phase transition. Zone c shows the stable regions where the heat dissipation of the substrate has no effect on tissue transformation; we found that in these regions, the grains appear as larger austenite grains and continuous dendritic residual ferrite. Zone d is the last layer, and its latter layers undergo no heat treatment, as compared to previous layers, which is in line with Figure 14d, which shows there are more ferrites in this layer.

#### 3.2.2. Microstructure at Junction

**Figure 15:**
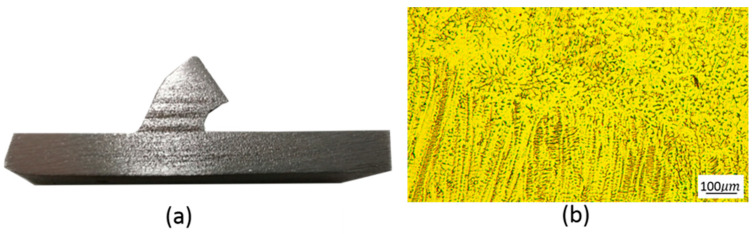
(**a**) Section of the connection between the rod and the lower panel; (**b**) Microstructure of the node.

Figure 15 is the sectional view and microstructure of the node with the face sheet. We can find that the inclined rod is fully connected to the lower panels without any air holes, which shows that WAAM has a huge advantage over traditional manufacturing methods. Figure 15b is the bonding area between the first layer and the lower substrate. We can see that there is more ferrite in this area than in Figure 14b. This can be explained by the fact that the heat dissipation effect of the substrate on the solder coating is gradually weakened from the substrate to the top of rod, which makes the cooling speed of the molten pool gradually slow down, and the diffusions of Cr, Ni and other alloy elements at the ferrite–austenite boundary are more sufficient when the ferrite is transformed into austenite, resulting in more austenite and less ferrite, as in Figure 14b. As Figure 15b shows, there are many ferrites in this area, and the main morphology is dense skeleton and flocculent.

### 3.3. Tensile Properties of the Rod

**Figure 16:**
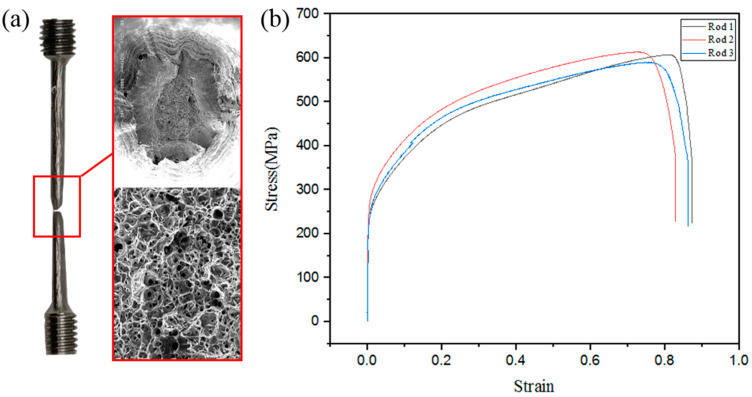
(**a**) Morphology of broken sample and SEM images of fracture surface; (**b**) Stress–strain curve of the tensile specimens.

In order to obtain the properties of rods, a tensile test involving three rods, fabricated by the parameters shown in Table 3 and machined to the size shown in Figure 6, was conducted. As is shown in Figure 16a, necking appeared in the sample, and there are many slip lines on the surface which are at an angle of 45° to the axis of the specimen. The 45° slip line is caused by the maximum shear stress. With the SEM images of fracture surface morphology, we can see that the fracture tearing edge is high and dimples are deep and numerous, which shows the good ductility of the rod. With three samples built under the same condition, we found that the average tensile strength of the inclined rods produced by WAAM with 304 L stainless steel is about 603 MPa, and the elongation-to-failure reaches 77%. Compared with the forging standards in ASTM A240/A240M-19 [31], the tensile strength of rods manufactured by WAAM exceeds the minimum forging standards by 24.3%. This can be explained by the manufacturing process. WAAM is a layer-by-layer accumulation of molten pool, and the frequency is low, which allows the molten pool to cool at a very fast rate. After each droplet has cooled down, the next one comes. Thus, the microstructure is fine, and the mechanical properties are high. This a great advantage of WAAM, compared with traditional casting and forging.

## 4. Conclusions

In this paper, large-scale pyramidal lattice structures were fabricated by WAAM. The manufacturing principle is presented in detail. The influences of two important parameters on forming, as well as molding defects, were investigated. What is more, the microstructure and the tensile properties of the inclined rod were analyzed. The main conclusions are given as follows:

1. The heat input and preset layer height are two parameters that control stable deposition. When the heat input is less than 5.53 J/mm, the welding arc force Fa is so low that the diameter of the rod is uneven, and some wire could not even be melted. When the heat input is more than 16.83 J/mm, the welding arc force Fa is so large that the molten pool will collapse. When the heat input is between 10.35 J/mm and 12.63 J/mm, we can get well formed rods, and their diameters increase as the heat input increases.
2. The microstructure of the rods is austenite and residual ferrite. The fusion line is clearly observed, which is perpendicular to the heat dissipation direction. The grains grow perpendicular to the fusion line in the opposite direction to the heat flow, with a morphology of dense skeleton and flocculent. Moving from the substrate to the top of rod, there is more austenite and less ferrite, and the grains grow larger. In the layer band, though, there are a lot of small residual linear ferrites at its grain boundary. The top of the rod is the last layer without reheating, and has a lot of worm-like ferrites.
3. The tensile strength of the inclined rods produced by WAAM with 304 L stainless steel is about 603 MPa, exceeding the forgings standards by 24.3%. The percentage elongation is 77%, which is very high, and exceeds the minimum elongation of forgings by 92.5%.

## Figures and Tables

**Table 1:** Actual chemical composition used in this experiment (wt.%).

Element	Fe	C	Mn	Si	S	P	Cr	Ni	Mo	Cu
Content	Rest	0.04	1.85	0.35	0.008	0.014	18.2	9.58	0.45	0.34

**Table 2:** Parameters of preheating.

Preheating Parameters	Value
Frequency, f (HZ)	0.5
Peak current, Ip (A)	100
Peak time ratio, β (%)	10
Base-to-peak current ratio, α (%)	5
Wire feed velocity, V (cm/min)	0
Preheating time, Tp (s)	6

**Table 3:** Four groups of different layer height.

	I	II	III	IV
Layer height, *H* (mm)	0.45	0.5	0.55	0.55 (first five layers)–0.5
Peak current, *Ip* (A)	100	100	100	100
Peak time ratio, *β* (%)	7	7	7	7
Base-to-peak current ratio, *α* (%)	5	5	5	5
Wire feed velocity, *V* (cm/min)	60	60	60	60

**Table 4:** Four groups’ deposition parameters concerning different heat inputs.

	I	II	III	IV
Peak current, Ip (A)	60	80	100	120
Peak time ratio, β (%)	5	6	7	8
Base-to-peak current ratio, α (%)	5	5	5	5
Average voltage, U (V)	12.7	16.1	17.3	18.7
First five layers height, Ho (mm)	0.7	0.63	0.55	0.35
Remaining Layer Height, Hr (mm)	0.67	0.58	0.5	0.3
Wire feed velocity, V (cm/min)	60	60	60	60

## References

[1] Nemat-Nasser S., Kang W.J., Mcgee J.D. (2007). Experimental investigation of energy-absorption characteristics of components of sandwich structures. Int. J. Impact Eng..

[2] Wadley H.N.G. (2006). Multifunctional periodic cellular metals. Philos. Trans. R. Soc. Lond. Ser. A Math. Phys. Eng. Sci..

[3] Evans A.G., Hutchinson J.W., Ashby M.F. (1998). Multifunctionality of cellular metal systems. Prog. Mater. Sci..

[4] Queheillalt D.T., Wadley H.N.G. (2005). Cellular metal lattices with hollow trusses. Acta Mater..

[5] Evans A.G., Hutchinson J.W., Fleck N.A., Ashby M.F., Walley H.N.G. (2001). The topological design of multifunctional cellular metals. Prog. Mater. Sci..

[6] Gibson L.J., Ashby M.F. (1997). Cellular Solids: Structure and Properties.

[7] Deshpande V.S., Fleck N.A., Ashby M.F. (2001). Effective properties of the octet-truss lattice material. J. Mech. Phys. Solids.

[8] Deshpande V.S., Ashby M.F., Fleck N.A. (2001). Foam topology: Bending versus stretching dominated architectures. Acta Mater..

[9] Lefebvre L.P., Banhart J., Dunand D. (2008). Porous Metals and Metallic Foams: Current Status and Recent Developments. Adv. Eng. Mater..

[10] Lee Y.H., Lee B.K., Jeon I., Kang K.J. (2007). Wire woven bulk Kagome truss cores. Acta Mater..

[11] Wadley H.N.G., Fleck N.A., Evans A.G. (2003). Fabrication and structural performance of periodic cellular metal sandwich structures. Compos. Sci. Technol..

[12] Kooistra G.W., Wadley H.N.G. (2007). Lattice truss structures from expanded metal sheet. Mater. Des..

[13] Li M., Wu L., Ma L., Wang B., Guan Z. (2011). Mechanical Response of All-composite Pyramidal Lattice Truss Core Sandwich Structures. J. Mater. Sci. Technol..

[14] Kooistra G.W., Deshpande V.S., Wadley H.N.G. (2004). Compressive behavior of age hardenable tetrahedral lattice truss structures made from aluminium. Acta Mater..

[15] Debroy T., Wei H.L., Zuback J.S., Mukherjee T., Elmer J.W., Milewski J.O., Beesea A.M., Wilson-Heid A., De A., Zhang W. (2018). Additive manufacturing of metallic components—Process, structure and properties. Prog. Mater. Sci..

[16] Gualtieri T., Bandyopadhyay A. (2018). Additive manufacturing of compositionally gradient metal-ceramic structures: Stainless steel to vanadium carbide. Mater. Des..

[17] Hamzah H.H.B., Keattch O., Covill D., Patel B.A. (2018). The effects of printing orientation on the electrochemical behaviour of 3D printed acrylonitrile butadiene styrene (ABS)/carbon black electrodes. Sci. Rep..

[18] Taufik M., Jain P.K. (2013). Role of build orientation in layered manufacturing: A review. Int. J. Manuf. Technol. Manag..

[19] Yan C., Hao L., Hussein A., Young P., Raymont D. (2014). Advanced lightweight 316L stainless steel cellular lattice structures fabricated via selective laser melting. Mater. Des..

[20] Gu D.D., Meiners W., Wissenbach K., Poprawe R. (2012). Laser additive manufacturing of metallic components: Materials, processes and mechanisms. Int. Mater. Rev..

[21] Williams S.W., Martina F., Addison A.C., Ding J., Pardal G., Colegrove P. (2016). Wire+Arc Additive Manufacturing. Mater. Sci. Technol..

[22] Zhenshu M., Guangsen C., Qianru W., Changmeng L., Yunfeng Z. (2018). Influence of Pulse Frequency and Heat Input on Macrostructure and Microstructure of TC4 Titanium Alloy by Arc Additive Manufacturing. Rare Met. Mater. Eng..

[23] Abe T., Sasahara H. (2019). Layer geometry control for the fabrication of lattice structures by wire and arc additive manufacturing. Addit. Manuf..

[24] Li Z., Liu C., Xu T., Ji L., Wang D., Lu J., Ma S., Fan H. (2019). Reducing arc heat input and obtaining equiaxed grains by hot-wire method during arc additive manufacturing titanium alloy. Mater. Sci. Eng. A.

[25] Xu T., Tang S., Liu C., Li Z., Fan H., Ma S. (2020). Obtaining large-size pyramidal lattice cell structures by pulse wire arcadditive manufacturing. Mater. Des..

[26] Guan K., Wang Z., Gao M., Li X., Zeng X. (2013). Effects of processing parameters on tensile properties of selective laser melted 304 stainless steel. Mater. Des..

[27] Yilmaz O., Ugla A.A. (2017). Microstructure characterization of SS308LSi components manufactured by GTAW-based additive manufacturing: Shaped metal deposition using pulsed current arc. Int. J. Adv. Manuf. Technol..

[28] ISO 6892-1:2009 (2009). Metallic Materials—Tensile Testing—Part 1: Method of Test at Room Temperature.

[29] Mettu S., Chaudhury M.K. (2011). Motion of Liquid Drops on Surfaces Induced by Asymmetric Vibration: Role of Contact Angle Hysteresis. Langmuir Acs J. Surf. Colloids.

[30] Karlsson L., Arcini H., Bergquist E.L., Weidow J., Börjesson J. (2010). Effects of Alloying Concepts on Ferrite Morphology and Toughness of Lean Duplex Stainless Steel Weld Metals. Weld. World Le Soudage Dans Le Monde.

[31] ASTM A240/A240M-19 (2019). Standard Specification for Chromium and Chromium-Nickel Stainless Steel Plate, Sheet, and Strip for Pressure Vessels and for General Applications.

